# The modified Glasgow prognostic score in Crohn’s disease—does it predict short-term outcome?

**DOI:** 10.1007/s10353-018-0518-0

**Published:** 2018-03-22

**Authors:** Stanislaus Argeny, Anton Stift, Michael Bergmann, Martina Mittlböck, Svenja Maschke, Stefan Riss

**Affiliations:** 1 Department of Surgery, Medical University of Vienna, Waehringer Guertel 18–20, 1090 Vienna, Austria; 2 Center for Medical Statistics, Informatics and Intelligent Systems, Medical University of Vienna, Spitalgasse 23, 1090 Vienna, Austria

**Keywords:** Modified Glasgow prognostic score, Crohn’s disease, Surgery, Complications, Anastomotic leakage

## Abstract

**Background:**

The modified Glasgow prognostic score (mGPS) has recently gained increased attention as a prognostic marker for malignant disease survival and postoperative short-term complications. Due to lacking data, the present study was conducted to correlate the mGPS with the postoperative course in patients following surgery for Crohn’s disease.

**Methods:**

We enrolled 341 patients who underwent intestinal resection for symptomatic Crohn’s disease at a tertiary referral centre between 2000 and 2014. All relevant data were obtained from the institutional database and individual chart review. Thirty-day morbidity was defined according to the Clavien–Dindo classification.

**Results:**

A total of 79 (23.17%) postoperative complications were identified (grade I and II: *n* = 54, 15.84%; grade III and IV: *n* = 23, 6.74%; grade V: *n* = 2, 0.59%). The mGPS did not show any correlation with an eventful postoperative course following surgery (no complication: median mGPS: 1, range 0–2; complications: median mGPS: 1, range 0–2; *p* = 0.8521). In addition, the occurrence of an anastomotic leakage was not associated with a higher mGPS (*p* = 0.8592). Patients with an acute indication for surgery (*n* = 29, 11.44%) had higher median mGPS (median: 2, range 0–2) in contrast to patients who were operated on electively (median: 1, range 0–2; *p* = 0.0003). No other correlation between surgical characteristics and mGPS was detected.

**Conclusions:**

In the present study, we could clearly demonstrate that an acute indication for surgery in symptomatic Crohn’s disease is associated with higher mGPS scores. However, the mGPS did not correlate with postoperative complications. Further studies are required to define the prognostic value of mGPS in Crohn’s disease patients.

## Introduction

Despite increased use of promising biological drug regimes, the treatment of Crohn’s disease (CD) remains challenging. Conservative treatment failure still occurs and the vast majority of affected patients will undergo intestinal resection at least once during their lifetime. Notably, patients with CD are at a higher risk to develop postoperative complications in comparison to patients without inflammatory bowel disease [1, 2]. Therefore, preoperative risk assessment is of crucial importance in order to choose the appropriate time for performing surgery and to minimize the probability of perioperative adverse events [3].

Identified risk factors for an eventful postoperative course are smoking, disease phenotype and emergency operation [4, 5], whereas the impact of immunosuppressive therapy and nutrition status on surgical outcome remains conflicting [6, 7].

Low level of albumin can be a result of various conditions, including a reduced nutrition status or a systemic inflammatory response. Recently, low preoperative serum albumin and elevated CRP (C-reactive protein) levels were found to be independent risk factors for intra-abdominal septic complications in CD [8].

The modified Glasgow prognostic score (mGPS), which includes both CRP and albumin, has gained increased attention as a prognostic instrument in oncological diseases. In fact, the mGPS has been proven to be a reliable prognostic marker for overall survival in patients with small cell lung cancer, colorectal malignancy and pseudomyxoma peritonei [9–11]. Notably, data regarding the role of mGPS as a marker for complications after surgery are lacking [12].

To our knowledge, this is the first study which aimed to assess the predictive role of preoperative mGPS for developing postoperative complications in patients undergoing intestinal resection for symptomatic CD.

## Materials and methods

We enrolled 341 patients (42.5% female) with a median age of 36.4 years, who underwent intestinal resection for symptomatic CD at a tertiary referral centre between 2000 and 2014. The study was approved by the ethics committee of the Medical University of Vienna and was performed in accordance with the ethical standards laid down in the 1964 Declaration of Helsinki and its later amendments.

Calculation of the modified Glasgow prognostic score is described in detail in Table 1 and uses preoperative values of CRP and albumin. The score ranges from 0 to 2 points, where a higher score indicates poorer prognostic outcome.

**Table 1:** Calculation of the modified Glasgow prognostic score (mGPS)

CRP	Albumin	mGPS
≤10 mg/L	≥35 g/L	0
≤10 mg/L	<35 g/L	0
>10 mg/L	≥35 g/L	1
>10 mg/L	<35 g/L	2

*mGPS* modified Glasgow prognostic score*, CRP* C-reactive protein

Preoperative albumin and CRP levels were derived from a peripheral blood sample. Patients who had no differential blood test available within 14 days prior to surgery were excluded from further analysis.

**Fig. 1:**
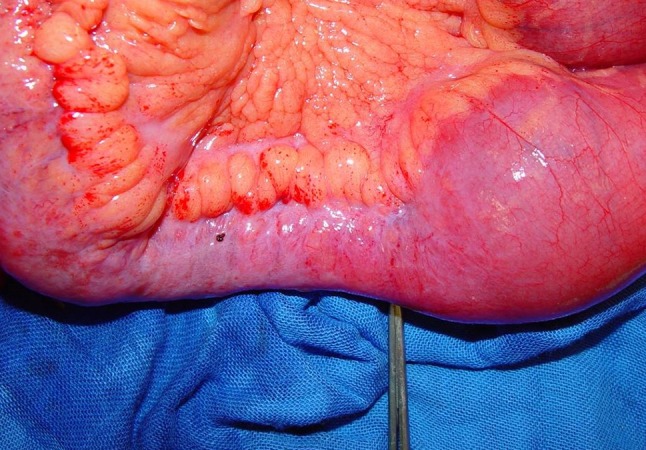
Crohn’s disease-related ileal stenosis

**Fig. 2:**
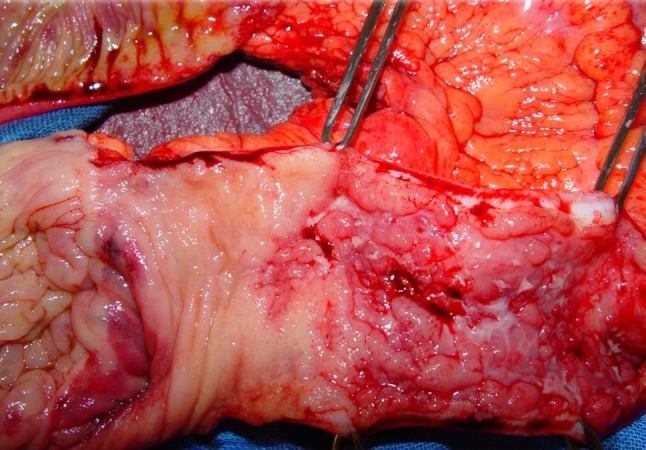
Surgical specimen showing Crohn’s disease typical cobblestone pattern

**Fig. 3:**
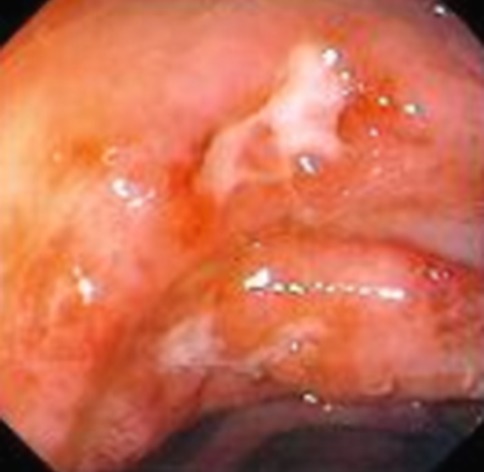
Endoscopic view of a colitis in Crohn’s disease

**Fig. 4:**
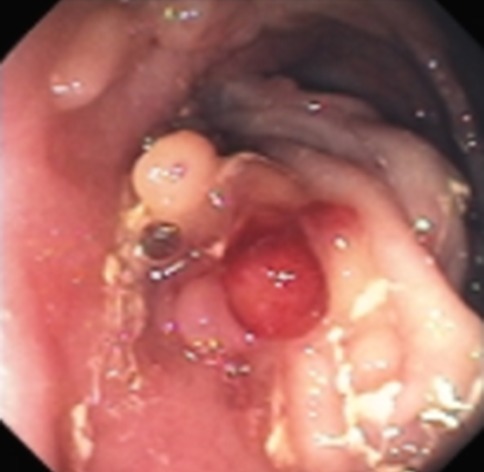
Endoscopic view of a jejunocolic fistula in Crohn’s disease

All operations were conducted or supervised by a single colorectal team specializing in the treatment of inflammatory bowel disease (Fig. 1, 2, 3 and 4). The laparoscopic approach has already been described previously in detail [4].

Demographic and clinical baseline data including information about surgical characteristics, blood tests and the use of immunosuppressive medications were obtained from the institutional database and individual chart review. Steroid therapy was defined as the use of corticosteroids until the day before surgery. Those patients who received steroids preoperatively but stopped treatment at least 2 days before surgery were regarded as “non-steroids” [13]. The exposure to azathioprin/6-mercaptopurin (AZA/6MP) was defined as the use of AZA/6MP within 14 days before operation. The administration of anti-TNF (TNF: tumor necrosis factor)antibody was recorded within 7 days before surgery.

Intestinal resections were divided into simple (1 intestinal resection) and complex (>1 intestinal resection). Thirty-day morbidity following surgery was described according to the Clavien–Dindo classification [14].

### Statistical analysis

Continuous data are shown as median, minimum and maximum due to skew distributions. Categorical variables are described with absolute numbers and percentages. In case of binary variables, monotone trends are tested by a trend version of the chi-square test. In case of sparse data, an exact chi-square test was calculated. All *p*-values were two-sided and *p* ≤ 0.05 was considered statistically significant. All calculations were performed with SAS version 9.3 (SAS Institute Inc., Cary, NC, USA).

## Results

Demographic and surgical characteristics are listed in Table 2 and 3.

**Table 2:** Demographic characteristics of patients operated on for Crohn’s disease

Demographic data		*N* or median (% or min–max)
Sex	Female	145 (42.5)
	Male	196 (57.5)
Age		36.4 (15.4–76.5)
BMI		21.1 (12.2–40.6)
Smoking	Yes	164 (48.1)
	No	177 (51.9)
Corticosteroids	Yes	282 (82.7)
	No	59 (17.3)
Anti-TNF antibody	Yes	13 (3.8)
	No	328 (96.2)
Azathioprine/6-mercaptopurine	Yes	74 (21.7)
	No	267 (78.3)

Data are described as *n* (%) or median (minimum–maximum)

*BMI* body mass index, *TNF* tumor necrosis factor

**Table 3:** Surgical characteristics in patients operated on for Crohn’s disease (CD)

Surgical characteristics			*N* (%)
Indication	Elective		302 (88.6)
	Acute		39 (11.4)
Surgical approach	Laparoscopic		136 (39.9)
	Laparotomy		173(50.7)
	Conversion		32 (9.4)
Type of resection	Simple (1 resection)		280(82.1)
	Complex (>1 resection)		61 (17.9)
Primary resection for CD	Yes		188 (55.1)
	No		153 (44.9)
Intraoperative findings	Stenosis	None	103(30.2)
		1	188 (55.1)
		>1	50 (14.7)
	Fistula	None	159 (46.6)
		1	117 (34.3)
		>1	65 (19.1)
	Inflammatory mass	None	193 (56.6)
		Yes	148 (43.4)
	Abscess	None	244 (71.6)
		Yes	97 (28.4)
Malignancy		None	334 (97.9)
		Yes	7 (2.1)
Perforating disease		No	161(47.2)
		Yes	180(52.8)

Data are described as *n* (%)

### Modified Glasgow score and surgical characteristics

Patients requiring an acute operation (*n* = 39, 11.4%) were found to have significantly higher median preoperative CRP levels (41.7 mg/L, range 4.6–304 vs. 13.4 mg/L, range 0.2–303.1; *p* = 0.0001) and lower median albumin levels (32.4 g/L, range 16.5–43.7) vs. 37.6 g/L, range 16.7–50.1; *p* = 0.0001) compared to patients who were operated on electively.

Similarly, patients with an acute indication for surgery (*n* = 29, 11.44%) had higher median mGPS scores (mGPS: 2, range 0–2) in contrast to patients, who were operated on electively (mGPS: 1, range 0–2), *p* = 0.0003.

No other correlation between surgical characteristics and mGPS was detected.

### Modified Glasgow score and postoperative complications

A total of 79 (23.2%) complications were observed and are outlined in Table 4. Thirty-day mortality was 0.6% (*n* = 2). One patient died due to cardiac failure following surgical revision for colonic ischemia after initial left hemicolectomy. A second patient died after developing an acute respiratory distress syndrome due to aspiration.

**Table 4:** Postoperative complications of patients operated on for Crohn’s disease according to the Clavien–Dindo classification

Clavien–Dindo classification	Complications	*N* (%)
Grade I (22)	Wound infections	6 (1.8)
	Paralytic ileus	14 (4.1)
	Other	2 (0.6)
Grade II (32)	Fever of unknown origin	11 (3.2)
	Other infection	4 (1.2)
	Urinal tract infection	3 (0.9)
	Pneumonia	1 (0.3)
	Others	6 (1.8)
	Paralytic ileus	7 (2.1)
Grade III (19)	Abscess	3 (0.9)
	Anastomotic dehiscence	11 (3.2)
	Mechanical ileus	1 (0.3)
	Deep wound infection	3 (0.9)
	Bleeding	1 (0.3)
Grade IV (4)	Pulmonary embolism	2 (0.6)
	Respiratory insufficiency	2 (0.6)
Grade V (2)	Multiple organ failure	1 (0.3)
	Circulatory failure	1 (0.3)

Data are described as *n* (%)

The median preoperative CRP level in patients who developed postsurgical complications was 13.4 mg/L (0.2 to 303.1), in contrast to a CRP value of 16.2 mg/L (0.4 to 304; *p* = 0.0998) in patients without complications. The preoperative albumin showed a median value of 36.3 g/L (16.7 to 50.1) in the complicated group, in comparison to 37.3 g/L (16.5 to 49.6) in the uncomplicated group (*p* = 0.1829).

The mGPS did not show a significant association with postoperative complications and major complications only (*p* = 0.8521 and *p* = 0.2333, respectively). The results are further outlined in Table 5.

**Table 5:** The modified Glasgow prognostic score in patients operated on for Crohn’s disease

		*N* (% relative)			*P*-value
mGPS		0	1	2	
Overall complications	None	95 (36.4)	92 (35.25)	74 (28.35)	0.8521
	Yes	35 (43.75)	18 (22.5)	27 (33.75)	
Major complications	None	116 (36.83)	105 (33.33)	94 (29.84)	0.2333
	Yes	14 (53.85)	5 (19.23)	7 (26.92)	

*mGPS* modified Glasgow prognostic score

### Modified Glasgow score and anastomotic leakage

In our series, a total of 11 patients (3.23%) developed an anastomotic leakage. Notably, median CRP and albumin levels were not significantly related to the development of anastomotic leaks (15.7 mg/L, range 2.4–133 vs.15.0 mg/L, range 0.2–304; and 39.5 g/L, range 28–50.1 vs. 37.0 g/L, range 16.5–49.6; *p* = 0.7282 and *p* = 0.1224, respectively). Concerning the mGPS, no correlation with anastomotic leakages were observed either (*p* = 0.8592).

## Discussion

In the present study, we aimed to investigate the predictive role of the mGPS on short-term outcome in patients following surgery for complicated CD. Although the mGPS was significantly elevated in patients undergoing acute surgical intervention, no impact on postoperative complications could be detected.

Preoperative CRP and albumin derived from peripheral blood samples are well-studied parameters for predicting surgical outcome. Heimann et al. showed in 126 patients undergoing surgery for CD that serum albumin lower than 35 g/L resulted in a 2.4-fold higher risk for non-septic complications [15]. Furthermore, Yamamoto et al. revealed in 540 operations for CD a preoperative albumin level lower than 30 g/L to be significantly associated with intra-abdominal septic complications [16]. In a more recent meta-analysis comprising 4189 operations for symptomatic CD, Hung et al. showed that low albumin levels increased the risk of abdominal complications 1.93-fold. However, the cut-off for defining low albumin varied among studies, thus available results need to be interpreted with caution [17]. In our series, the threshold for hypoalbuminemia was <35 g/L. Notably, those patients, who developed complications had median albumin levels above 35 g/L, which might also explain our non-significant findings.

In a recent multicentre study, Frasson et al. included 1102 patients who underwent right hemicolectomy for malignant disease. The authors found albumin significantly decreased in patients developing anastomotic leakages (35 g/L, range 30–40 vs. 39 g/L, range 35–43) [18]. We observed 11 patients with anastomotic leakage, but serum albumin levels of these patients (39.5 g/L, range 28–50) were comparable with the non-leakage group as reported by Frasson et al.

Additionally, Qin et al. conducted a retrospective study and found the C‑reactive protein/albumin ratio to be suitable to monitor disease activity in CD patients [19]. In another investigation from China, Zou et al. demonstrated that preoperative CRP >10 mg/L was an independent risk factor for intra-abdominal sepsis complication, defined as anastomotic leakage, intra-abdominal abscess, or enterocutaneous fistula within 1 month after surgery [8]. Three hundred forty-four CD patients had a primary anastomosis, of whom 39 patients (11.34%) developed an intra-abdominal septic complication with the need for reoperation in 12 cases. Preoperative serum albumin lower than <35 g/L was significantly associated with the occurrence of an eventful postoperative course (33.33 vs.14.43%). These findings are in contrast to our results, where median albumin levels showed no differences in patients experiencing major complications and those who did not. The anastomotic leakage rate was reported to be 4.65% and therefore slightly higher compared to our findings (3.23%). Additionally, only 39 cases (11.34%) were operated on laparoscopically, compared to 136 (39.9%) in our study.

Low serum albumin might not only reflect poor nutrition status, but also a critical overall condition with a state of chronic or acute inflammation [20–22]. The mGPS was first defined by McMillan et al., who revealed that the preoperatively calculated mGPS significantly correlates with overall and cancer-specific survival in colorectal cancer patients [10]. Moreover, other studies confirmed the superior role of the combined measurement of albumin and CRP, displayed by the mGPS, to predict postoperative outcome in various oncological entities [9, 23, 24].

Watt et al. examined the role of postoperative mGPS in predicting postoperative outcome in patients undergoing colorectal resection for malignancy [12]. Notably, patients who developed postoperative complications had significantly higher scores compared to those without.

Interestingly, mGPS in CD has not been addressed so far, although this particular group often presents malnourished and with elevated inflammatory markers.

A few limitations need to be addressed. As the study was conducted retrospectively, selection bias cannot be ruled out completely. We accepted blood tests within 14 days prior to surgery only. Although unlikely, CRP and albumin could have changed shortly before the date of operation.

## Novel aspects

- First study to assess the predictive preoperative value of the mGPS in symptomatic Crohn’s disease.
- Patients requiring emergency operation show higher mGPS values.
- Preoperative mGPS did not correlate with postoperative adverse events.

## Conclusion

In the present study we could show that an acute indication for surgery in symptomatic CD is associated with higher mGPS scores. Notably, preoperative mGPS did not have a predictive role for an eventful postoperative course in CD. Further studies are required to define the prognostic value of mGPS in CD patients.

## References

[1] Bernell O, Lapidus A, Hellers G (2000). Risk factors for surgery and postoperative recurrence in Crohn’s disease. Ann Surg.

[2] Iesalnieks I, Dederichs F, Kilger A, Schlitt HJ, Agha A (2012). Postoperative morbidity after bowel resections in patients with Crohn’s disease: risk, management strategies, prevention. Z Gastroenterol.

[3] Kristo I, Stift A, Bergmann M, Riss S (2015). Surgical recurrence in Crohn’s disease: are we getting better?. World J Gastroenterol.

[4] Riss S, Bittermann C, Zandl S (2010). Short-term complications of wide-lumen stapled anastomosis after ileocolic resection for Crohn’s disease: who is at risk?. Colorectal Dis.

[5] Indar AA, Young-Fadok TM, Heppell J, Efron JE (2009). Effect of perioperative immunosuppressive medication on early outcome in Crohn’s disease patients. World J Surg.

[6] Crowell KT, Messaris E (2015). Risk factors and implications of anastomotic complications after surgery for Crohn’s disease. World J Gastrointest Surg.

[7] Yamamoto T, Spinelli A, Suzuki Y (2016). Risk factors for complications after ileocolonic resection for Crohn’s disease with a major focus on the impact of preoperative immunosuppressive and biologic therapy: A retrospective international multicentre study. United European Gastroenterol J.

[8] Zuo L, Li Y, Wang H (2015). A practical predictive index for intra-abdominal septic complications after primary anastomosis for Crohn’s disease: change in C‑reactive protein level before surgery. Dis Colon Rectum.

[9] Fan H, Shao ZY, Xiao YY (2016). Comparison of the Glasgow Prognostic Score (GPS) and the modified Glasgow Prognostic Score (mGPS) in evaluating the prognosis of patients with operable and inoperable non-small cell lung cancer. J Cancer Res Clin Oncol.

[10] McMillan DC, Crozier JE, Canna K, Angerson WJ, McArdle CS (2007). Evaluation of an inflammation-based prognostic score (GPS) in patients undergoing resection for colon and rectal cancer. Int J Colorectal Dis.

[11] Tan GH, Novo CA, Dayal S (2017). The modified Glasgow prognosis score predicts for overall and disease-free survival following cytoreductive surgery and HIPEC in patients with pseudomyxoma peritonei of appendiceal origin. Eur J Surg Oncol.

[12] Watt DG, McSorley ST, Park JH, Horgan PG, McMillan DC (2017). A postoperative systemic inflammation score predicts short- and long-term outcomes in patients undergoing surgery for colorectal cancer. Ann Surg Oncol.

[13] Argeny S, Stift A, Mittlbock M (2016). Advanced age impacts surgical characteristics and postoperative course in patients with Crohn’s disease. Int. J. Surg..

[14] Dindo D, Demartines N, Clavien PA (2004). Classification of surgical complications: a new proposal with evaluation in a cohort of 6336 patients and results of a survey. Ann Surg.

[15] Heimann T, Greenstein A, Mechanic L, Aufses A (1984). Early complications following surgical treatment for Crohn’s disease. Ann Surg.

[16] Yamamoto T, Allan RN, Keighley MR (2000). Risk factors for intra-abdominal sepsis after surgery in Crohn’s disease. Dis Colon Rectum.

[17] Huang W, Tang Y, Nong L, Sun Y (2015). Risk factors for postoperative intra-abdominal septic complications after surgery in Crohn’s disease: a meta-analysis of observational studies. J. Crohns Colitis.

[18] Frasson M, Granero-Castro P, Ramos Rodriguez JL (2016). Risk factors for anastomotic leak and postoperative morbidity and mortality after elective right colectomy for cancer: results from a prospective, multicentric study of 1102 patients. Int J Colorectal Dis.

[19] Qin G, Tu J, Liu L (2016). Serum albumin and C‑reactive protein/albumin ratio are useful biomarkers of Crohn’s disease activity. Med Sci Monit.

[20] Dominguez de Villota E, Mosquera JM, Rubio JJ (1980). Association of a low serum albumin with infection and increased mortality in critically ill patients. Intensive Care Med.

[21] Fleck A, Raines G, Hawker F (1985). Increased vascular permeability: a major cause of hypoalbuminaemia in disease and injury. Lancet.

[22] Fuhrman MP, Charney P, Mueller CM (2004). Hepatic proteins and nutrition assessment. J Am Diet Assoc.

[23] Shafique K, Proctor MJ, McMillan DC (2013). The modified Glasgow prognostic score in prostate cancer: results from a retrospective clinical series of 744 patients. BMC Cancer.

[24] Ni XC, Yi Y, Fu YP (2015). Prognostic value of the modified glasgow prognostic score in patients undergoing radical surgery for hepatocellular carcinoma. Medicine (Baltimore).

